# *Neospora caninum* and Wildlife

**DOI:** 10.5402/2013/947347

**Published:** 2013-06-24

**Authors:** Sonia Almería

**Affiliations:** Departament de Sanitat i Anatomia Animals and Centre de Recerca en Sanitat Animal (CreSA), Universitat Autònoma de Barcelona Bellaterra, 08193 Barcelona, Spain

## Abstract

Bovine neosporosis caused by *Neospora caninum* is among the main causes of abortion in cattle nowadays. At present there is no effective treatment or vaccine. Serological evidence in domestic, wild, and zoo animals indicates that many species have been exposed to this parasite. However, many aspects of the life cycle of *N. caninum* are unknown and the role of wildlife in the life cycle of *N. caninum* is still not completely elucidated. In North America, there are data consistent with a sylvatic cycle involving white tailed-deer and canids and in Australia a plausible sylvatic cycle could be occurring between wild dogs and their macropod preys. In Europe, a similar sylvatic cycle has not been established but is very likely. The present review is a comprehensive and up to date summary of the current knowledge on the sylvatic cycle of *N. caninum*, species affected and their geographical distribution. These findings could have important implications in both sylvatic and domestic cycles since infected wildlife may influence the prevalence of infection in cattle farms in the same areas. Wildlife will need to be taken into account in the control measures to reduce the economical losses associated with this important disease in cattle farms.

## 1. Introduction

*Neospora caninum* is an obligate intracellular protozoan first described in dogs in 1984 [1] and identified since then in a wide range of warm-blooded animals, including many wildlife species [2–4]. Before 1988, when Dubey et al. [5] described a new genus and species, *N. caninum,* this parasite was probably misdiagnosed as *Toxoplasma gondii* [5]. Nowdays *N. caninum* is considered to be one of the main causes of abortion in cattle worldwide [4].

The control and prophylaxis measures necessary for bovine neosporosis, already complex, will be further complicated if wildlife plays a role in the domestic and sylvatic cycle of the parasite, as seems likely. 

The knowledge of the role of wildlife species as reservoirs of *N. caninum* and its implications in the cycle of this parasite is increasing, and many studies have been reported to date. The present review is a comprehensive analysis of present knowledge on the sylvatic cycle of *N. caninum* and summarizes the studies of presence of specific antibodies, DNA detection and isolation of *N. caninum* in wildlife to date, species affected, and their geographical distribution. Previous reviews [2–4] described prevalence levels from many studies, therefore, details on specific prevalence levels will only be given for the most recent studies.

## 2. Life Cycle and Transmission of *Neospora caninum*

*Neospora caninum* has a wide host range (ruminants such as bovine, goats, sheep, or water buffalo, equids, and carnivorous species, including many wild species), but it is primarily a disease of cattle and dogs. Serological results in multiple species, including domestic, wildlife, and zoo animals provide evidence that many species have been exposed to this parasite. For detailed information on host range and geographic distribution in wildlife to date, see Tables 1–7. Another species, *N. hughesi*, classified on the basis of unique molecular and antigenic characteristics has been identified only in horses [6] and is not included in this review. 

Although there is a concern about the zoonotic potential of *N. caninum*, there is no compelling proof that *N. caninum* successfully infects humans (reviewed [3, 7]). 

In the life cycle of *N. caninum*, there are three known infectious stages: tachyzoites and bradyzoites in tissue cysts are found intracellular in the intermediate hosts in which asexual replication takes place, while oocysts are excreted by the definitive hosts in which sexual replication occurs. Bradyzoites are morphologically similar to tachyzoites but are found inside tissue cysts which are round or oval in shape and can reach more than 100 microns. They are essentially found in the central nervous system although they have also been described in other tissues which include muscles of naturally infected dogs and calves [8]. The wall of the cyst is thick (up to 4 microns), smooth, and devoided of septa; it is positively stained by the PAS stain (reviewed by [3]).

*Neospora caninum* can be transmitted transplacentally (also termed vertically, congenitally, or endogenous transplacental transmission) and postnatally (also termed exogenous transplacental transmission [9]). Postnatal transmission occurs by ingestion of tissues infected with tachyzoites or tissue cysts and/or by ingestion of food or drinking water contaminated with sporulated oocysts. During an acute phase of infection, tachyzoites may be found in virtually all host tissues and fluids, including peripheral blood, placenta, and amniotic fluid of pregnant cows [10, 11]. When tachyzoites reach brain tissues, they may differentiate into bradyzoites probably due to the immune response against the protozoan, resulting in formation of tissue cysts [8]. The reactivation of bradyzoites into tachyzoites, mostly in pregnancy, will result in vertical transmission from the dam to its foetus [3]. 

Vertical transmission is considered the main route of transmission in cattle and other domesticated Bovidae species such as the water buffalo (*Bubalus bubalis*) [12], and it also seems to be frequent in wildlife ruminants. For example, very high (approximately 85%) seropositive of *N. caninum* in fawns suggests a high rate of congenital transmission of the parasite in white-tailed deer [13]. However, the importance and the incidence of vertical transmission in maintaining infection in many wild species remain unknown.

Domestic dogs and some wild canids are the only known definitive host of *N. caninum* able to excrete oocysts. In domestic dogs, oocysts are excreted in an unsporulated stage [14, 15] and sporulate outside the host in as few as 24 hours [15]. It is unclear how long sporulated oocysts can survive in the environment, but appear to be very resistant [3]. In dogs, oocysts production occurs from 1 day to 27 days after ingestion of intermediate host tissues such as infected mouse or calf tissues [14, 16, 17]. Dogs shed low numbers of oocysts for a transient period, but in one study, relapse of shedding was observed in dog faeces collected at an interval of 4 months [18]. It is unknown if the shedding is continuous or the dog resheds the oocysts due to reinfection. In wild canids little is known of oocyst excretion duration and relapses, but in coyotes experimentally infected with tissues, one of four coyotes shed approximately 500 *Neospora*-like oocysts between 8 and 10 days after infection [19]. 

To date, dogs (*Canis lupus familiaris*) [14], coyotes (*C. latrans*) [19], dingoes (*C. lupus dingo*) [20], and more recently, gray wolves (*C. lupus lupus*) [21] have been confirmed as definitive hosts for *N. caninum, *being able to excrete environmentally resistant oocysts. Other wild canids present in each different country will need to be studied for their potential role as definitive hosts [2]. 

The ingestion of oocysts is the only demonstrated mode for horizontal transmission in herbivores [3]. To date, cow-to-cow transmission of *N. caninum* has not been observed. Neonatal calves may become infected after ingestion of milk contaminated with tachyzoites [22], and *N. caninum* DNA in milk, including colostrum, has been demonstrated [23]. However, there is no conclusive proof that lactogenic transmission of *N. caninum *occurs [17]. There is no evidence that venereal transmission can occur, although the presence of the parasite in semen has been demonstrated [24, 25]. 

As indicated previously, the main transmission route of *N. caninum* in cattle and possible the main route in wild ruminants is vertical transmission from the dam to its foetus. However, although congenital transmission can be high, vertical transmission by itself cannot perpetuate *N. caninum* infection in cattle herds [10, 26, 27]. Therefore, a point exposure of cattle, and other ruminants, to *N. caninum* oocysts has to take place. Such point exposure of cattle to *N. caninum* oocysts has been implicated in abortion storms [28, 29], and wild canids may be important in this regard.

### 2.1. Established and Putative Sylvatic Cycles of *N. caninum*

Many aspects of the life cycle of *N. caninum* are still unknown and the role of wildlife in the life cycle of *N. caninum* is still not completely elucidated, but a sylvatic cycle between domestic and wild canids and ruminants is thought to be important in the biology of *N. caninum* [14, 19].

Since it has been possible to identify seropositive cattle on farms where no domestic dogs were present, this has led to the question whether canids other than dogs might be involved in the horizontal transmission of *N. caninum* [30, 31]. 

Early studies already hypothesized with the idea of a sylvatic cycle. Ferroglio et al. [32] reported that herbivores diggers and pure grazer species showed a higher seroprevalence than browser animals, and they suggested that this could be due to a great exposure to *N. caninum* oocysts shed on pastures by definitive hosts. The same authors suggested that in Kenya wild carnivore species could act as the *N. caninum* definitive host considering the absence of infection in feral and rural dogs in the area of the study [32]. In addition, the hypothesis of a transmission between wild canids and beef cattle was already shown to be epidemiologically consistent by Barling et al. [33]. These authors considered the presence of coyotes and gray foxes *(Urocyon cinereoargenteus)* a risk factor for transmission of *N. caninum* to beef cattle [33]. At that time this was only a hypothesis, but subsequent studies have shown that the hypothesis was correct. 

In North America, there is data consistent with a sylvatic cycle of *N. caninum* between cervids and canids, involving the white tailed-deer and domestic canids [34, 68]. White-tailed deer have shown elevated seroprevalence of infection in several studies (reviewed by Dubey et al., [35]), viable *N. caninum* has been isolated from this host [36], and, more importantly, dogs shed *N. caninum* oocysts after being fed with the brain of naturally exposed white-tailed deer [68]. The later authors, in order to determine whether deer could transmit *N. caninum*, fed 4 dogs with brains of naturally infected white-tailed deer (*Odocoileus virginianus*), and 2 of these dogs shed oocysts. Then the named NC-deer1 oocysts were administered to a calf that developed a high antibody titer, providing evidence that *N. caninum* from wildlife can infect cattle. 

In Australia, a plausible sylvatic cycle could be occurring between wild dogs (including dingoes) and their macropod prey [37]. Experimental infections of the fat-tailed dunnart (*Sminthopsis crassicaudata)*, a carnivorous marsupial, have provided indirect support for the existence of a sylvatic life cycle of *N. caninum* in this country [38]. 

A similar sylvatic cycle among wild ruminants and wild canids seems probable in Europe but has not been established yet. A study conducted in Hungary observed that farm dogs that were seropositive to *N. caninum* often ate aborted or dead calves or consumed raw offal of game animals including deer [39]. 

In Europe, red foxes (*Vulpes vulpes*) are the main wild canid species, and there are small populations of gray wolves (*Canis lupus*). Reports of antibodies in red foxes are very numerous (reviewed by Sobrino et al. [40]) and *N. caninum* DNA has been demonstrated in the brains of red foxes in several European countries (see Section 4.1.1(2-(b)). Although *N. caninum*-like oocysts were found in the faeces of free-ranging red foxes in Canada [41], to date, red foxes have not been proven to be a definitive host of the parasite by experimental inoculation [42], and the examination of a sylvatic cycle with foxes as definitive hosts and deer, roe deer, and wild mice analysis has yielded no evidence indicating that the examined animals were part of a sylvatic cycle for *N. caninum *in Germany [31].

## 3. Diagnostic Tools for Detection of *Neospora caninum*-Infected Animals in Wildlife

Detection of antibodies to *N. caninum* is a good indicator of exposure of animals to the parasite. For diagnosis of *N. caninum* in live domestic animals, particularly in cattle, detection of antibodies in milk or serum has been shown to be the best option both at the herd and the individual level. In cattle, a great variety of assays are available for serologic analysis of *N. caninum* such as indirect immunofluorescent antibody test (IFAT), *Neospora *agglutination test (NAT), enzyme-linked immunosorbent assay (ELISA), immunoblotting, or western blot (WB) [4, 43, 44]. Of those, the ELISA test is very commonly used, and several commercial ELISA tests are available (e.g., see comparison of techniques by von Blumröder et al. [45]). The IFAT is a well-established technique for detecting anti-*N. caninum* antibodies in different animal species. Antibodies to other protozoans such as *T. gondii*, *Sarcocystis* spp., and *Babesia canis* do not cross-react with *N. caninum* tachyzoites in the IFAT at dilutions of 1 : 50 or higher [46].

Serological analysis in wildlife is more complicated. Firstly, blood sample collection is not easy and in some instances, has logistic, ethical, and economic difficulties [47]. Frequently most of the samples are collected postmortem and some can be old, and degradation of immunoglobulins could have taken place. In addition, assays are more limited since species-specific secondary antibody and conjugates are often not available. The majority of studies in wildlife rely on competitive ELISA techniques (cELISA) and agglutination tests (NAT) because they do not need species-specific secondary antibodies. For competitive ELISAs, the principle of competition makes this test theoretically possible to be used in any other species but validation data are not yet available for many species. Most of the tests have only been validated for bovine and dog sera, and the specificity, sensitivity and cutoff value of serological tests have not been evaluated in many wild species. Therefore, confirmation of the results by other tests should be implemented. 

In cattle, serological results by different techniques have shown good agreement [44, 45], and in wild ruminants in Spain, an excellent agreement between cELISA and IFAT was observed [48]. Seropositive sera from different noncarnivorous wildlife species by a commercial ELISA *N. caninum* monocupule screening kit from laboratories Pourquier (P00510/02) (France) (validated for bovine sera) were confirmed by IFAT. Of the 35 positive samples in the screening ELISA, 32 were also found positive by IFAT, with a kappa value of agreement between both serological tests of 0.929. When only data from red deer were included in the agreement analysis, the kappa value was 0.920. 

Wolf et al. [49] using the immunoblot as reference technique found that IFAT exhibited a sensitivity and specificity of about 95% analysing *N. caninum* antibodies in sera from South American camelids, and Dubey et al. [13] analyzed antibodies in white-tailed deer by four different techniques including IFAT (cut-off 1 : 25), NAT (cut-off 1 : 25), an ELISA and WB and found that the majority of animals were positive in all of them. Antibodies to *N. caninum* were found in 150 of 170 (88.2%) by any of the 3 tests (99 by western blots, 135 by ELISA, 106 by IFA, and 118 by NAT). 

However, when carnivorous species are analyzed, poor test agreement has been observed, and some studies have shown that the tests did not classify the same animals as seropositive [40, 66]. Sobrino et al. [40] analyzed multiple species sera by cELISA (VMRD, Pullman, WA, USA) screening and confirmatory NAT and/or IFAT and observed very low agreement between ELISA and IFAT in red foxes (*Vulpes vulpes*), although better agreement between both techniques was observed in Iberian lynxes (*Lynx pardinus*). Higher detection of antibodies was achieved by cELISA, especially compared to IFAT that could be due to the antigens utilized by both techniques. While IFAT uses whole tachyzoite antigen, that expose only surface antigens, ELISA uses sonicated tachyzoite that could expose both internal and surface antigens. Wapenaar et al. [66] observed that IFAT showed an excellent sensitivity with control samples, but the seroprevalence detected in natural samples from red foxes and coyotes (*Canis latrans*) were the lowest in comparison with other assays (ELISA, NAT and Immunoblotting). It also needs to be taken into account that in IFAT there is a subjective assessment of the observed fluorescence.

Silva et al. [50] observed the same seropositivity to *N. caninum* (8.5% of 59 samples) in captive maned wolves (*Chrysocyon brachyurus*) from Brazil using homologous and heterologous fluorescent conjugates. However, Sobrino et al. [40] could not confirm cELISA positive samples in European wolves using IFAT with a heterologous conjugate, while NAT showed high agreement with cELISA. 

Recently, King et al. [51] in order to estimate *N. caninum* prevalence in aboriginal community dogs and wild dogs from Australia used a new optimized cutoff of 18.5% inhibition for the cELISA (VMRD, Pullman, WA, USA), previously validated for use in cattle and partially validated for use in dogs, and which has as recommended cut-off a value greater than 30% percentage inhibition (%I). These authors calculated the new cut-off using a two-graph receiver-operating characteristic (TG-ROC) analysis and IFAT as the gold standard resulting in an equal sensitivity and specificity of only 67.8% in canids. 

Since serological analysis in wild carnivores is complex and can be influenced by poor quality of the sera tested one has to be careful interpreting individual data evaluated with only one serological assay [40, 44]. According to some authors, when only blood is available, immunoblotting should be used as an additional test to IFAT, ELISAs, and agglutination tests [2]. Other authors use a combination of a minimum of two different positive serological methods to confirm seropositivity in the animals [40]. 

Applying a serological test to fluids collected from dead wild animals instead of blood has been performed in some studies [47, 52], and some authors consider, this could be an alternative to sera samples in wildlife [47]. Murphy et al. [52] examined thoracic fluid (pleural fluid and clotted blood) from 220 thoracic fluid red fox samples for *N. caninum* antibodies using IFAT. A total of six (3%) foxes had antibodies to *N. caninum*. Jakubek et al. [47] analyzed antibodies to *T. gondii *by agglutination (*Toxo*-screen test) (DAT) in pleural fluid and lung extract collected from 56 carcasses of red foxes and found that antibodies were still detectable in the same fluids kept at room temperature for 28 days, although in fewer foxes, which according to the authors indicated the potential utility of using fluids from carcasses for antibody screening of wild animals at the population level [47], as could also be the case for *N. caninum*. 

In addition, another potential confounding factor when performing serology for *N. caninum* in wild animals is infection with *Hammondia heydorni*, the closest phylogenetically related protozoan parasite to *N. caninum* (reviewed by [2, 53]). Serological tests for *H. heydorni* are not yet available. *Hammondia heydorni* also has a canid-ruminant life cycle [53] and dogs and other canids, such as red foxes and coyotes, may serve as definitive host for *H. heydorni*. Sporulated oocysts are infective for cattle, sheep and goats, which may serve as intermediate hosts (IH) and its oocysts appear morphologically similar to those of *N. caninum*. It is possible that serological cross-reactions can take place between *N. caninum* and *H. heydorni*, so it is necessary to be cautious when identifying new hosts of *N. caninum* in wildlife on the basis of only antibody detection [2]. Molecular techniques are available to differentiate *N. caninum* and *H. heydorni* infections [2].

PCR-based diagnosis has become an excellent method for confirmation of *N. caninum* in infected tissues and to isolate parasite stages in many species. Most studies focus on brain samples, since this organ is the most frequent location of the parasite in chronically infected animals [3]. Recent studies have shown that detection by PCR in several species only in samples from brain may potentially lead to an underestimation of the levels of *N. caninum* infection in IH species. Bartley et al. [54] observed positive samples in badgers, ferret, and polecats in tissues other than brain, such as heart and skeletal muscle, while similarly, PCR positive samples in skeletal muscles have been reported in rodents [55].

## 4. Seroprevalence, Prevalence, and Isolation Studies of *Neospora caninum* in Wildlife 

The present review shows data on seroprevalence and, when available, on DNA detection and isolation in the different wildlife species reported to date. Since data on seroprevalence levels vary among countries and locations and most of the data are not comparable due to different techniques and different cutoffs used, the present review only shows the species affected and the geographical distribution of positive records. It includes all positive studies of presence of antibodies of *Neospora caninum* on wildlife species published to date, independently of the techniques used. Since several of these studies were based on only one serological technique not validated for the wild species involved in the study, some of these results should be considered with caution. 

The first wild ruminants reported to be infected or seropositive to *N. caninum* were mostly cervids: *N. caninum *tissue cysts were found in the brain of a full-term stillborn Eld's deer (*Cervus eldi siamensis*) in Paris Zoo [56], and antibodies were observed in black and white tailed deer (*Odocoileus hemionus and O. virginianus*) [57, 58] in the USA, and in roe deer (*Capreolus capreolus*), red deer (*Cervus elaphus*) (both cervids), and in chamois (*Rupicapra rupicapra*) (in the Bovidae family) in Europe [59]. In wild carnivore species, the first species reported to be seropositive to *N. caninum* were canids: coyote (*Canis latrans*) [60], red fox (*Vulpes vulpes*) [61], and gray fox (*Urocyon cinereoargenteus*) [62]. These data already gave an indication of the importance of these species in the sylvatic cycle of *N. caninum*. Since then, antibodies against *N. caninum* and, in some cases *N. caninum* DNA, have been reported in multiple species which suggests that these species could also act as hosts of the parasite in nature.

### 4.1. *Neospora caninum* Studies in Carnivores

As carnivores are at the top of the food chain, measuring their prevalence of *N. caninum* can give an indication of the presence of *N. caninum* infections lower down the food chain [62–64]. The level of *N. caninum* infection acquired by a carnivore will vary depending on the IH consumed [3, 64]. Therefore, by investigating which aspects of carnivore's ecology are associated with the variation of prevalence between and within different species, we can further improve our understanding of carnivore's role in the epidemiology of neosporosis [64]. 

#### 4.1.1. *Neospora caninum* Studies in Wild Canids

Canids are important in the epidemiology of *N. caninum* infection because they are the only hosts reported to date that can excrete the environmentally resistant oocysts. Reports on seroprevalence are of interest because, as occurs with *T. gondii*, seropositive animals might have already shed *N. caninum* oocysts in the environment. In addition, as indicated previously, surveys of *N. caninum* infection in free-ranging canids can provide an estimation of environmental contamination and circulation of *N. caninum* in domestic and wild ecosystems.

*(1) Confirmed Definitive Hosts (DHs)*

*(a) Coyotes. *North American coyotes (*Canis latrans*) were the first wild canid confirmed to be DHs of *N. caninum* [19]. Four captive-raised coyote pups consumed tissues from *N. caninum*-infected calves. Their faeces were examined from 4 days before to 28 days after infection. One pup shed *N. caninum*-like oocysts, which tested positive for *N. caninum* and negative for *H. heydorni* using PCR tests [19]. Historically, coyotes were confined to the prairie areas of North America, but during the last 100 years the coyotes' geographical range has expanded to include the entire continental USA, much of Canada, and Central America (reviewed by [19]). The expanding range and population of these animals has increased the probability of contact with domestic animals, and this increases the risk of *N. caninum* transmission between coyotes and livestock. In Texas, Barling et al. [33] performed a spatial analysis study and found statistical associations among the density of cattle, seropositivity for *N. caninum*, and abundance of coyotes and gray foxes (*Urocyon cinereoargenteus*). Since then several studies have shown positive presence of *N. caninum* antibodies with different seroprevalence levels in this species in the USA [19, 60, 65] and Canada [66] (Table 1). 

*(b) Australian Dingoes.* Australian dingoes (*Canis lupus dingo) *were also confirmed as DHs of *N. caninum *[20]. Three dingo pups raised in captivity were fed tissue from calves infected with an Australian isolate of *N. caninum*, Nc-Nowra. Oocysts of *N. caninum*, confirmed by species-specific PCR, were shed in low numbers by one dingo pup at 12–14 days postinfection (p.i.). The remaining animals did not shed oocysts. Furthermore, the blood from two out of three dingoes tested positive for DNA of *N. caninum* using PCR tests at 14 and 28 days p.i. Oocyst shedding from a dingo demonstrates that dingoes are DHs of *N. caninum* and horizontal transmission of *N. caninum* from dingoes to farm animals and wildlife may occur in Australia [20]. Large mammals, predominantly macropods, are the main food source for dingoes, with small mammals including rodents, forming a minor part of dingo diets (reviewed by King et al. [51]). Although dingoes were identified as DHs of *N. caninum*, only a single study of prevalence of antibodies against *N. caninum* in wild dingo populations in Australia has been reported [67].

Canids in Australia also include wild dogs and Aboriginal community dogs. Of those, wild dogs consist of the dingo, feral domestic dog (*C. lupus familiaris*), and their hybrid genotypes [51]. In a recent study, a high seroprevalence of *N. caninum* was observed in wild dogs and Aboriginal community dogs. Of the 263 dog sera tested, the true prevalence of *N. caninum* antibodies was 27.0% by cELISA and IFAT (95% confidence limit: 10.3–44.1%), and the authors postulated that the populations of free-ranging dogs are likely to be important contributors to the sylvatic life cycle of *N. caninum* [51]. 

*(c) Wolves.* The gray wolf (*Canis lupus lupus*) is the most recent wild canid confirmed as a natural DH for *N. caninum* to date [21]. The discovery was made in the USA. *Neospora*-like oocysts were found microscopically in the faeces of three of 73 wolves from Minnesota examined at necropsy. *N. caninum*-specific DNA was amplified from the oocysts of all three wolves. Oocysts from one wolf were infective for gamma interferon gene knockout (KO) mice, and viable *N. caninum* (designated NcWolfUS1) was isolated in cell cultures seeded with tissue homogenate from the infected mouse. This observation suggests that wolves may be an important link in the sylvatic cycle of *N. caninum *[21].

In Europe, some populations of wolves still live in certain countries. In a study in Spain, Sobrino et al. [40] observed that Iberian wolves *(Canis lupus signatus)* had the highest seroprevalence of infection in the Canidae family (21.4% of 28 wolves), higher than that observed in red foxes. The seroprevalence in wolves in the study was lower than the seroprevalence observed by Gondim et al. [68] in North America (39% of 164 wolves), but higher than the observed in Alaskan wolves by Dubey and Thulliez [69] (3.3% of 122 wolves). This data could indicate an important role of this species in the epidemiology of *N. caninum* in the areas where wolves are found in Spain but needs to be confirmed. The differences in *N. caninum* seroprevalence in wolves and red foxes could be related to their diet. While wolf diet is mainly based on ruminants, red foxes are omnivorous. 

In a recent study, *N. caninum* antibodies were analyzed in 109 wolves in Scandinavia [70] by iscom ELISA, and those with absorbance values exceeding 0.20 were also analysed by immunoblotting. Four (3.7%) wolves were positive. From one male wolf, three samples were collected over a 7-year period. No antibodies were detected at the first sampling in 1998 when it was approximately 8 months old but when it was sampled again 5 and 7 years later the ELISA and immunoblotting were positive [70].

In Israel, very low seroprevalence was observed, but presence of antibodies was found in one wolf of 9 analyzed with an IFAT titer of 1 : 400 [71].

(*2*) *Other Canids as Possible Definitive Hosts (DHs)*

(*a*)* Wild Maned Wolves (Chrysocyon brachyurus). *To date other species of wild canids have not been confirmed as new DH. The presence of *N. caninum* antibodies has been reported in captive wild maned wolves, which are not included in the genus *Canis* (see Section 4.1.3 on *N. caninum* in wild carnivores in captivity and zoo collections, Table 2). Maned wolves are the largest canids in South America and according to the IUCN list [72] are a near-threatened species. Several studies reported negative results in this species in Brazil [73–75]. 

*(b) Foxes. *Red foxes (*Vulpes vulpes*) are the main wild canid species in Europe. As indicated previously, although, presence of oocysts has been observed in naturally-infected red foxes in Canada [41], to date, red foxes have not been proven to be DHs of the parasite by experimental inoculation [42]. In Europe, reports of antibodies in red foxes are very numerous (reviewed Sobrino et al. [40]) and *N. caninum*-DNA has been demonstrated in the brains of red foxes in Catalonia, North-East Spain [76], the Czech Republic [77] and more recently in Belgium [78], in Ireland [64] and in Great Britain [54]. In an early study, Murphy et al. [52] in Ireland observed that six (3%) red foxes had antibodies to *N. caninum *but PCR assays carried out on DNA extracted from the 33 brains with histological lesions were negative for *N. caninum. *However, a recent study reported the presence of *N. caninum* DNA in red foxes in Ireland [64]. 

The levels of infection observed in red foxes by PCR are not high: 10.7% of 122 red foxes in Spain [76]; 4.61% of 152 in Czech Republic [77], 6.6% of 304 brain samples in Belgium [78], 6% of 156 red foxes in Ireland [64], and 4.8% of 83 red foxes in Great Britain [54]. Similarly, most studies have shown low seroprevalence of *N. caninum* in red foxes in Europe. Antibodies in Ireland and Britain were only 3% and 0.9% [52, 79], respectively. Very low titres (≤1 : 40) were observed in 4.4% of 45 red foxes in Poland [80] and low levels were also observed (3.2% of 95 red foxes) in some areas of Spain [40]. Negative results were reported in Sweden [63]. The current data would appear to indicate that foxes are not involved in a sylvatic cycle for *N. caninum* in Europe [54]. A recent survey found no evidence of parasite DNA in brain samples from over 500 red foxes, and there was no evidence indicating that foxes, deer, roe deer and wild mice were part of a sylvatic cycle in Germany [31]. However, very high seroprevalence in red foxes was observed in an area of Catalonia [81], indicating localized *N. caninum* infection among areas and countries as occurs with other wildlife species [40, 48, 81]. Therefore, the lack of infection on one country or area does not preclude sylvatic cycle in another country or area within a country.

Many other species of foxes in North and South America have been reported to be seropositive to *N. caninum *(Tables 1 and 2). So far none has been described as a DH of *N. caninum*. There are very few studies in areas other than Europe and America. In one study in Israel, 1 of 24 (4.2%) red foxes analyzed had antibodies to *N. caninum* [71]. 

*(c) Golden Jackals (Canis aureus)*. Golden jackals are included in the genus *Canis, *and morphological and molecular studies indicate a greater affinity to the gray wolf and coyote than to jackal so they could be possible DHs of *N. caninum*. The golden jackal is indigenous to North and north-eastern Africa, south-eastern and central Europe (up to Austria and Hungary), Asia Minor, the Middle East and Southeast Asia. To our knowledge, only one study has reported antibodies to *N. caninum* in golden jackals. Of 114 free-ranging wild golden jackals analyzed in Israel, only two showed IFAT titres of 1 : 50 [71].

(*d*)* African Wild Dogs (Lycaon pictus). *A recent study has shown high seroprevalence in African wild dogs (*Lycaon pictus*) (52% of 87 African wild dogs sampled by IFAT ≥ 1 : 40), much higher than that compared to domestic dogs in the same study (18% of 6) in Kenya [82]. This species is a canid only found in Africa. 

#### 4.1.2. Carnivorous Intermediate Hosts (IHs)

The species of carnivore noncanids with reported antibodies against *N. caninum* to date together with the countries in which have been reported are indicated in Table 1. 

*(1) Mustelids, Viverrids, and Herpestids. *Mustelids are carnivores whose main diet are small mammals, mainly rodents and lagomorphs, and birds, although they have also been known to eat fruits, invertebrates, carrion, and garbage (reviewed by Sobrino et al. [40]). Several years ago, ermine or stoats (*M. erminea*), weasels (*M. frenata*), and ferrets (*M. putorius*) were tested to determine if they could be DHs of *N. caninum* being fed *N. caninum*-infected mice, but oocysts were not observed, so the hypothesis was not supported in these species in the USA [83]. 

Several mustelid species have been reported seropositive to *N. caninum *in Europe. In Spain, *N. caninum* antibodies in mustelids have been shown in Eurasian badgers *(Meles meles*), stone martens* (Martes foina*), pine martens (*M*.* martes*), and polecats (*Mustela putorius*) by both cELISA and IFAT [40]. In that study, although differences were not statistically significant among the different sampled taxonomic families (Canidae, Felidae, Viverridae, Herpestidae and Mustelidae), the highest prevalence of *N. caninum* was observed in mustelids (14.5%). This fact has been previously observed by Sedlák and Bártová [84] in zoo animals from the Czech-Republic analyzed by IFAT, although in different species to those analyzed in the study of Sobrino et al. [40]. DNA of the parasite was not detected in 88 mustelids in the same country by Hůrková and Modrý [77], but very recently, two studies have reported presence of *N. caninum* DNA in species of mustelids in Ireland and the UK. In Ireland from 221 American minks (*Mustela vison*  syn  *Neovison vison*) analyzed, antibodies were observed in 1% and presence of DNA in 3%. No antibodies or DNA was observed from 60 pine martens, 51 badgers, 41 stoats and 4 feral ferrets (*Mustela furo*) [64]. On the other hand, Bartley et al. [54] analyzed *N. caninum* in brain and other tissues from wild carnivores in Great Britain and observed parasites by PCR in ferrets (10/99, 10.1%), polecats (13/70, 18.6%), American mink (3/65, 4.6%), and Eurasian badgers (7/64, 10.9%). No parasites were detected in stoats (0 of 9). The PCR results from this study, along with antibody data [40] have demonstrated that several mustelid species are infected with *N. caninum* when they encounter the parasite in the environment [54]. 

In Herpestids, Egyptian mongooses (*Herpestes ichneumon*) showed *N. caninum* antibodies but only when assessed by cELISA and could not be confirmed by IFAT [40]. Similarly, presence of antibodies was also observed in the Viverrid, common genet (*Genetta genetta*), by cELISA but could not be confirmed by IFAT [40].

*(2) Wild Felids and Wild Cats. *In Spain, *N. caninum* antibodies in wild felids have been reported in Iberian lynx (*Lynx pardinus*), in European wildcats *(Felis silvestris silvestris*) [40, 85], and in feral cats (*Felis silvestris catus*) [86]. The Iberian lynx is critically endangered [72], with approximately 250 individuals currently inhabiting only two isolated metapopulations in southern Spain (Sierra Morena and Doñana) [40]. Antibodies to *N. caninum* were observed in 12.0% of 25 Iberian lynx (*Lynx pardinus*) [40]. The European wildcat (*Felis silvestris silvestris*) is categorized as vulnerable in Spain. These wildcats are found in a wide variety of habitats, primarily associated with forests with low density of humans. Antibodies to *N. caninum* were observed in 16.7% of 6 European wildcats (*Felis silvestris silvestris*) [40]. 

Feral cats (*Felis silvestris catus*) are the other type of wild felid present in Spain. In a study in Mallorca, Balearic Islands, Spain of 59 feral cats (captured in baited traps during authorized predator control campaigns), seroprevalence to *N. caninum*, assayed by cELISA (VMRD) and confirmed by IFAT was low (6.8%, 4 of 59) [86]. 

Sedlák and Bártová [84] observed antibodies in Eurasian lynxes (*Lynx lynx*) in zoos from the Czech Republic. The presence of *N. caninum* antibodies has also been reported in several free-living wild feline species such as lions and cheetahs [32, 87] in Africa (Table 1). In addition, many felids have shown antibodies against *N. caninum* in zoos (see Table 2). Felids most probably, only act as IHs in neosporosis. After oral inoculation of cats with tissue cysts of *N. caninum* faecal shedding of oocysts was not observed [88]. So far no clinical cases of *N. caninum* have been described in naturally infected felids, although an experimental study in domestic cats showed *N. caninum* infection in immunocompromised and immunocompetent animals (revised by Sobrino et al. [40]). 

*(3) Australian Carnivore Marsupials. *In Australia, a sylvatic life cycle of *N*. *caninum *has been hypothesized between dingoes and small marsupials and rodents [37]. Experimental infections of the fat-tailed dunnart (*Sminthopsis crassicaudata*), a carnivorous marsupial widely distributed throughout the arid and semiarid zones of Australia, showed that this species can act as an IH for *N. caninum *[38]. In addition, dunnarts offer a new animal model in which active neosporosis is dominated by tissue cyst production. An unprecedented number of cysts were observed to be widespread in the dunnart's musculature [38]. The high susceptibility of marsupials to *N. caninum *observed in this study could parallel their susceptibility to *T. gondii *[38]. Oocysts were not observed in the dunnarts in the study.

*(4) Neospora caninum in Carnivore-Scavenger Wild Birds.* Birds may be another reservoir host for wild dogs or even domestic dogs [89]. Recently *N. caninum* has been demonstrated in a few species of naturally infected birds, in particular in domestic chicken (*Gallus domesticus*), in sparrows (*Passer domesticus*) [89–91], and recently in scavenger-carnivorous wild birds from Spain, DNA has been detected in magpies (*Pica pica*) and in the common buzzard (*Buteo buteo*) [92]. The presence of birds on cattle farms has been related to outbreaks of abortion and proposed as a risk factor for *N. caninum *infection (reviewed by Molina-López et al. [93]). Birds have not been confirmed as DHs of *N. caninum* and their exact role in *N. caninum* cycle is unknown. A possibility is that birds could carry oocysts and help in the dissemination of *N. caninum* oocysts in the environment.

High *N. caninum* seroprevalence has been observed in crows (*Corvus corax*) trapped in farms suffering abortions in Catalonia, Spain [93]. In this study antibodies to *N. caninum* were found in 24 (35.8%; IC 95%: 24.5–48.5) of 67 common ravens tested by IFAT with titres ranging from 1 : 50 (*n* = 18) to ≥1 : 100 (*n* = 6). The high seroprevalence detected suggest a role for this species in the epidemiology of *N. caninum* [93].

#### 4.1.3. *Neospora caninum* Studies in Wild Carnivores in Captivity and Zoo Collections

There have been several studies of seroprevalence of *N. caninum* in carnivores in zoos. Sedlák and Bártová [84] detected antibodies in many carnivore species in zoos in the Czech Republic which included Eurasian wolf (*Canis lupus*), maned wolf (*Chrysocyon brachyurus*), Chiloe fox (*Pseudalopex fulvipes*), cheetah (*Acinonyx jubatus*), jaguarundi (*Puma yagouaroundi* syn.* Herpailurus yagouaroundi*), Eurasian lynx (*Lynx lynx*), Indian lion (*Panthera leo goojratensis*), fisher (*Martes pennanti*) and fennec (*Vulpes zerda) *[84]. 

Mattos et al. [94] reported for the first time presence of antibodies in captive bush dogs (*Speothos venaticus)* in Brazil. Presence of antibodies in this species was also reported more recently in zoos from the same country by André et al. [95]. In addition, these later authors detected antibodies in the following wild carnivore species in zoos from Brazil: ocelot (*Leopardus pardalis*), little spotted cat (*Leopardus tigrinus*), jaguar (*Panthera onca*), puma, (*Puma concolor*), jaguarandi, tiger (*Panthera tigris*), Pampas cat (*Oncifelis colocolo*), caracal (*Caracal caracal*), serval (*Letailurus serval*), lion (*Panthera leo*), fishing cat (*Prionailurus viverrinus*), crab-eating fox (*Cerdocyon thous*), maned wolf, hoary fox (*Pseudalopex vetulus*), and European graywolf [95]. 

Antibodies have been reported in farm-breed blue foxes (*Alopex lagopus*) in China [96]. Other studies of presence of *N. caninum* antibodies in captive and zoo species are indicated in Table 2.

### 4.2. *Neospora caninum* Studies in Herbivores

Nowadays, *N. caninum *is considered one of the main causes of abortion and stillbirth in cattle worldwide [4]. The effect of *N. caninum* infection on wild herbivores, in particular ruminants, is the focus of the many studies that are reviewed here.

#### 4.2.1. Studies in Wild Ruminants

With few exceptions, *N. caninum* has been reported in different species of wild herbivores in the individual continents. Therefore, the species of wild ruminants positive for the presence of antibodies, DNA, or for isolation of the parasite are presented by continent (Table 3).

Like cattle, wild ruminant species can only get infected through ingestion of sporulated oocysts in water or feed, and/or by transplacental transmission. Several studies have implied that vertical transmission, as occurs in cattle, is also the main route of transmission of *N. caninum* in wild ruminants. To date congenital or vertical transmission has been reported in Eld's deer (*Cervus eldi siamensis*) in a Paris Zoo [56], in fallow deer (*Dama dama*) [97] and recently, in white-tailed deer (*Odocoileus virginianus*) [35]. Neosporosis was diagnosed in full-term stillborn twin calves of captive antelopes (*Tragelaphus imberbis*) in a German zoo. In both calves a multifocal nonsuppurative encephalitis was present and infection with *N. caninum* was confirmed by foetal serology and PCR [98]. In free-ranging caribou (*Rangifer tarandus*) in Alaska, a study compared antibodies to *N. caninum* in young versus adult animals and the results suggested that vertical transmission may also be an important component of new infections in the Alaskan caribou [65]. Similarly, the very high (84.9%) seropositivity of *N. caninum* in white-tailed deer (WTD) fawns [13], suggested a high rate of congenital transmission of the parasite in this species. In other wild species the importance and incidence of vertical transmission in maintaining *N. caninum* infection remains unrecognised.

*(1) American Wild Ruminants.* The species of wild ruminants reported as possible IHs of *N. caninum* in America include white-tailed deer, black-tailed deer, caribou, moose (*Alces alces*), American bison (*Bison bison*), musk ox (*Ovibos moschatus*), elk (*Cervus canadensis*), mule deer (*Odocoileus hemionus hemionus*), and Pampas deer (*Ozotoceros bezoarticus*) (Table 3).

In the USA, WTD is considered one of the most important wildlife reservoirs of *N. caninum,* and a sylvatic cycle involving WTD and canids was confirmed in this country [68]. The parasite has also been isolated in this species [36]. Numerous studies have indicated the importance of WTD in the epidemiology of neosporosis in the USA (Table 3). In the most recent study, Dubey et al. [13] tested *N. caninum* antibodies in sera from white-tailed deer from Minnesota and Iowa by four serologic tests including IFAT, NAT, an ELISA, and WB and observed very high seroprevalence in both states. Of 62 adult deer from Minnesota antibodies to *N. caninum* were found in 44 (71%), and in Iowa, antibodies to *N. caninum* were found in 150 of 170 WTD (88.2%) by any of the 3 tests [13]. More recently, the presence of antibodies in WTD has been reported in Northern Mexico [112]. In this study the overall prevalence for *N. caninum* was 8.4% (31/368 WTD) tested by ELISA (IDEXX) [112]. 

Systemic neosporosis was reported in a California black-tailed deer [57]. In this species, Dubey et al. [116] observed *N. caninum* antibodies in 8 of 43 black-tailed deer in the USA. However, the most recent study did not find seropositive animals in Alaska (0% of 55 black-tailed deer analyzed) [65]. In the same study,* N. caninum* antibodies were observed in free-ranging caribou [65] with a seroprevalence of 11.5% of 453 caribous analyzed by IFAT, which was higher than that observed for *T. gondii* [65]. 

Another recent study in Western Canada has shown *N*. *caninum* positive results in 25% of 20 elk, 75% of 20 WTD, 5% of 20 caribou, and 10% of 20 moose analyzed by IFAT [115]. 

*(2) European Wild Ruminants*. The species of wild ruminants reported as possible IHs of *N. caninum* in Europe include red deer (*Cervus elaphus*), roe deer (*Capreolus capreolus*), chamois (*Rupicapra pyrenaica*), Alpine ibex (*Capra ibex*), Spanish ibex (*Capra pyrenaica hispanica*), European mouflon (*Ovis musimon *syn. *O. aries*), fallow deer (*Dama dama*), Barbary sheep (*Ammotragus lervia*), moose, and European bison (*Bison bonasus bonasus*  L.) (Table 3). 

*Neospora caninum* has been recently isolated from naturally infected European bison [127]. Surprisingly, the isolate was achieved from peripheral blood. The authors loaded the white blood cells from two strongly positive and two negative bison on monolayer Vero cells culture and observed viable tachyzoites only in the positive samples at days 60 and 70 after incubation. The tachyzoites were evaluated by PCR and sequence analysis, and the isolate was subsequently named NC-PolBb1 and NC-PolBb2. Prior to this study, the presence of antibodies to *N. caninum* in European bison was reported by Cabaj et al. [128] also in Poland, and antibodies were found in European bison in zoos from the Czech Republic by Sedlák and Bártová [84] (Table 4). 

In some European countries, comprehensive surveys have examined antibodies to *N. caninum* in wild ruminants. For example, in Spain, antibodies by cELISA and confirmatory IFAT have been observed in wild ruminants such as red deer, roe deer, Barbary sheep [48, 122], and Spanish Ibex [125]. Negative results were observed in 79 fallow deer, 27 European mouflon, 40 chamois, and 3 Spanish ibex by Almería et al. [48]. In the most recent study of wild ruminants in Spain, García-Bocanegra et al. [125] observed that 30 of 531 (5.6%) Spanish ibex had antibodies to *N. caninum* using a cELISA, of which 27/30 (5.1%) were confirmed as seropositive by IFAT. 

In the Czech Republic, Bártová et al. [123] analyzed 720 wild ruminants for antibodies to *N. caninum* by screening cELISA and confirmatory IFAT. *N. caninum* antibodies were found in 14% (11 positive of 79 tested) roe deer, 14% (2 of 14) sika deer (*Cervus nippon*), 6% (24 of 377) red deer, 1% (2 of 143) fallow deer, 3% (3 of 105) European mouflon, and neither of 2 reindeer (*Rangifer tarandus*). 

Numerous studies have shown *N. caninum* seroprevalence and/or infection in European cervids, particularly in red deer and roe deer (Table 3). To our knowledge, the parasite has not been isolated from either of these two species to date. In recent studies, in Poland, of 47 free-living red deer analyzed by iscom ELISA and those samples exceeding 0.400 absorbance units analyzed by WB, 6 sera were positive by both techniques [119], and in Greece, of 60 wild deer 5% were seropositive to *N. caninum* [120].

Low seroprevalence levels were observed in recent studies in roe deer: 2.7% of 73 roe deer analyzed in Belgium [78], and from 199 roe deer analyzed by iscom ELISA and confirmed by WB only 1 roe deer was positive by WB, (0.5%) and regarded as *N. caninum* positive in Sweden [124]. However, importantly, *N. caninum* was found in brain samples from the 2 roe deer in Belgium. The presence of *N. caninum* DNA confirms this species as a natural IH of *N. caninum* and seems to indicate that roe deer might be an important wild ruminant species in the epidemiology of *N. caninum* in Europe. The roe deer is a small-sized cervid (subfamily Odocoileinae) abundant throughout Europe and in some countries, such as Spain its population is increasing [156].

Negative results were reported from 4 fallow deer and 7 red deer in Belgium [78] and from 417 moose sampled in Sweden [124], where (4.1%) iscom ELISA positive samples could not be confirmed by WB [124].

A fatal case of meningoencephalomyelitis caused by *N. caninum* was diagnosed in a juvenile fallow deer (*Dama dama*) in a zoo in Switzerland [97]. Antibodies against *N. caninum* in this species were not found in Spain [48] or Belgium [78] but have been observed in the Czech Republic [123] although at a low percentage (1% of 143 animals). Recently, in farmed fallow deer in Poland, a low seroprevalence (2.9% of 335 farmed fallow deer analyzed) was also observed using a cutoff value in the ELISA test (IDEXX) of optical density exceeding 0.159 absorbance units and confirmation by WB [126]. 

In addition to red deer and roe deer, *N. caninum* antibodies have been reported in Alpine chamois (*Rupicapra rupicapra*) and in Alpine ibex (*Capra ibex ibex*) in the Italian Alps [59, 118, 121]. On the other hand, in a recent study, samples from 651 Alpine ibex from 14 colonies throughout the Swiss Alps were negative for *N. caninum* [157]. 

Some of the above reports of wild ruminants in Europe included farmed deer. The study by Bártová et al. [123] in the Czech Republic included wild and captive ruminants, and Bień et al. [126] analyzed farmed fallow deer from Poland. However, in Poland, Goździk et al. [119] compared farmed versus free-living red deer, and the results of seroprevalence were very similar (13% of 47 free-living red deer and 11% of 106 farmed red deer were seropositive to *N. caninum*, resp.).

*(3) Asian Wild Ruminants and Herbivores*. A recent report has added a new deer species to the list of IHs of *N. caninum *from Asia. Meng et al. [129] reported the presence of *N. caninum* antibodies in 8.0% (17 of 218) Tarim red deer (*Cervus elaphus yarkandensis*) from Xinjiang Province, Northwest China by cELISA. Also in China, *N. caninum* antibodies were reported in red panda (*Ailurus fulgens)* in a zoo [131] (Table 4). Red pandas eat mostly bamboo, but may eat small mammals, birds and eggs. In Japan, sika deer were analyzed with negative results [158]. 

*(4) African Wild Ruminants and Herbivores. *Compared to the domesticated water buffalo (*Bubalus bubalis*), which behave in many ways like cattle, few studies of *N. caninum* infection have been performed in the wild African buffalo (*Syncerus caffer*). The presence of antibodies was observed in Kenya by Ferroglio et al. [32] and in zoo collections by Sedlák and Bártová [84]. In Kenya, many other species of wild ruminants had antibodies against *N. caninum* in the same study [32] (Table 3). 

#### 4.2.2. *Neospora caninum* in Herbivore Marsupials

Marsupials are very susceptible to *T. gondii* infection, and it might be expected that marsupials acquiring *N. caninum *would succumb to the disease at a high rate [38]. However, reports of *N. caninum* in marsupials to date are rare. Only recently a case of neosporosis was reported in a captive Parma wallaby (*Macropus parma*) from a zoo in Austria (Table 4) which died suddenly and was subjected to a necropsy examination. The main finding was necrotizing myocarditis associated with protozoan parasites and the protozoa were identified as *N. caninum* by use of immunohistochemistry and partial gene sequence analysis [136]. Further work is required to determine whether marsupials are an accidental or terminal host of this protozoan in order to better understand the host-parasite relationship [136].

#### 4.2.3. *Neospora caninum* Studies in Captive and Zoo Collection Wild Herbivores

Numerous ruminants have been found seropositive to *N. caninum* in zoos. In the Czech Republic, Sedlák and Bártová [84] detected antibodies in blackbuck (*Antilope cervicapra*), lechwe (*Kobus leche*), African buffalo, eland (*Taurotragus oryx*), European bison, sitatunga (*Tragelaphus spekei gratus*), Pere David's deer (*Elaphurus davidianus*), Thorold's deer (*Cervus albirostris*), Eastern elk (*Cervus elaphus canadensis*), and Vietnam sika deer (*Cervus Nippon pseudaxis*). Antibodies in Vietnam sika deer were also reported by Bártová et al. [123] in animals in captivity in the Czech Republic. 

Recently, Wiengcharoen et al. [132] reported detection of *N. caninum* antibodies in captive elephants (*Elephaus maximus indicus*) in Kanchanaburi Province (Thailand) with high seroprevalence (33.04% of 115 elephants by cELISA). Of those, only 7/115 (6.1%) were positive for both *N. caninum* and *T. gondii*. Surprisingly, a higher seroprevalence for *N. caninum* was observed in elephants compared to the prevalence of *N. caninum* infection in dairy cattle in Thailand from prior studies [132].

Three different clinical cases were described in white rhinoceros (*Ceratotherium simum*) (order Perissodactyla which includes horses). In one case, death in a young white rhinoceros in a game-breeding centre was reported by Williams et al. [133] in South Africa. In a second case, a 16-year-old female white rhinoceros died suddenly without clinical signs in a zoo in Thailand [134]. Histopathological analysis revealed disseminated protozoan tachyzoites in the liver, adrenal cortex, kidney, and intestine, and the organism was identified as *N. caninum* by immunohistochemistry and PCR. In addition, a third case of abortion, confirmed by PCR, was reported in a white rhinoceros in a zoo in Australia by Sangster et al. [135]. This species appears to be particularly susceptible to *N. caninum* infection.

#### 4.2.4. *Neospora caninum* in Lagomorphs and Rodents

*(1) Hares and Wild Rabbits. *Hares are considered a highly susceptible species to *T. gondii* infection in Europe. Acute generalized toxoplasmosis has been confirmed as the cause of death in European brown hares (*Lepus europaeus*) and mountain hares (*Lepus timidus*) in several studies (reviewed by Fernández-Aguilar et al. [159]). Little is known of the susceptibility of hares to *N. caninum*. A recent study observed a higher seroprevalence of *N. caninum* antibodies in brown hares in the Czech Republic and Austria compared to that for *T. gondii* [140], which could indicate that hares survive *N. caninum* infection in nature and may be less susceptible to *N. caninum* than to *T. gondii* infection. In this study, antibodies against *N.  caninum*  were observed in 129 (39%) of 333 hares from the Czech Republic, in 143 (37%) of 383 hares in Austria and in 8 (4%) of 209 hares in Slovakia, analyzed by cELISA [140]. Mixed infections (concurrent presence of both *N. caninum* and *T. gondii* antibodies) were found in 25 (8%) hares in the Czech Republic and in 14 (4%) hares in Austria and were absent in hares in Slovakia [140].

Rabbits have been shown to be a natural IH of *N. caninum* by molecular methods [138]. *Neospora caninum* infection prevalence was 10.5% (6/57), and 8.8% (5/57) of wild rabbits were coinfected with both *N. caninum* and *T. gondii *[138]. Investigation of tissue distribution determined that *N. caninum* DNA was most often detected in the brain and heart, less often in the tongue, and was not detected in the liver [138]. In Europe, Almería et al. [48] did not detect antibodies in any of the 251 wild rabbits in Spain, while low seroprevalence was observed in Iberian hares (*Lepus granatensis*). Low seroprevalence was also reported in European hares imported to Italy from East Europe [139]. 

In farm rabbits in northern Egypt, Ibrahim et al., [137] observed antibodies to *N. caninum* in only one sample (1.85%) by an ELISA using surface antigen 1 of *N. caninum* (NcSAG1t ELISA).

*(2) Rodents*. Rodents around farms have been shown to be a plausible IH of *N. caninum*, with demonstration of *N. caninum* DNA in feral rats and mice. However, although *N. caninum* has been documented frequently in tissues of asymptomatic rodents, viable parasites have not been isolated [4]. 

 The species of rodents in which *N. caninum* DNA has been detected include the field or wood mouse (*Apodemus sylvaticus*), rat (*Rattus norvegicus*), house mouse (*Mus musculus*), capybara (*Hydrochaeris hydrochaeris*), common vole (*Microtus arvalis*), and water vole (*Arvicola terrestris*) (reviewed by Dubey and Schares, [4]). Very recently, *N. caninum* has been reported in rock squirrel (*Spermophilus variegates*) [149] and, on organic farms in the Netherlands, DNA was reported in harvest mouse (*Micromys minitus*) (15.4%) and in two species of insectivores: the common shrew (*Sorex araneus*) (33.3%), and white-toothed shrews (*Crocidura russula*) (10.8%) [150]. The same authors also reported DNA in wood mice (17.6%) and common voles (4.2%) (Table 5).

In an area relatively free of cats, Thomasson et al. [143] observed a low *N. caninum* prevalence in field mice (3.4%, 95% CI: 0.12%–6.66%) and house mice (3.1%, 95% CI: 0.11%–6.05). While the presence of the parasite in rodents in relation with farms has been reported in several studies, DNA of *N. caninum* was not detected in any of the samples of *Rattus rattus*, *Rattus norvegicus*, and *Mus musculus* captured in urban areas of São Paulo in Brazil [160]. 

The possibility that dogs could be infected by eating infected house mice suggests new opportunities for *N. caninum* prophylaxis and control [55]. In addition, ingestion of dead rodents and insectivores by farm animals, either by accident (ruminants) or on purpose (pigs) could lead to the transmission of the parasite [150]. The fact that small mammals such as rodents and insectivores could easily be harbouring the parasite makes them a candidate for a good indicator species for parasitic contamination on farms [150].

The presence of *N. caninum* antibodies was observed in capybaras in two studies in Brazil [145, 146]. In the most recent study [146] 3% of the serum samples from 63 capybaras were positive. In addition, the parasite was found, by molecular analysis, in the lymph nodes, heart, liver, and blood of 23% of 26 capybaras (*Hydrochaeris hydrochaeris*) studied in the same country [147]: an indication of the importance of this rodent in the countries where it is common. 

#### 4.2.5. Clinical Cases in Herbivores

Few clinical cases have been reported in wild herbivores. Most cases are reported in captive animals, since clinical cases and abortion in free-ranging ruminants would be more difficult to observe in natural conditions. As indicated previously, these cases included systemic fatal neosporosis in non-pregnant adult black-tailed deer as reported by Woods et al. [57]; foetal infection in Eld's deer in zoos in France [56]; fatal cases in captive antelopes in zoos in Germany [98], fatal cases in white rhinoceros in zoos in South Africa, Thailand, and Australia [133–135]. Recently, another fatal case of neosporosis was reported in a captive Parma wallaby in a zoo in Austria [136].

### 4.3. *Neospora caninum* in Omnivores

Omnivorous can acquire *N. caninum* by ingestion of tissue cysts from other IHs, sporulated oocysts in feed and water, and transplacentally. 

The presence of antibodies against *N. caninum* has been observed in omnivore species such as wild boars which frequently eat rodents or other small mammals in the wild (Table 6). Although no recent studies have been reported, earlier reports showed antibodies to *N. caninum* in 102 (18.1%) of 565 wild boars in the Czech Republic analyzed by cELISA and confirmed by IFAT [151]. Mixed infection was found in 38 wild boars [151]. In Spain, *N. caninum* was sporadically (0.3% of 298) observed in wild boar (*Sus scrofa) *analyzed by the same techniques (cELISA and confirmatory IFAT) [48]. In another suidae Species, the warthog (*Phacochoerus aethiopicus*), antibodies were reported by Ferroglio et al. [32] in Africa (Table 6).

In a different group of omnivores,* N. caninum* antibodies have been reported in 84 of 396 South American opossum (*Didelphis marsupialis*) analyzed from the city of São Paulo, Brazil [152], while on the other hand, 30 North-American opossum (*Didelphis virginiana*) were seronegative to *N. caninum* in the USA [161]. American opossums are small-to medium-sized marsupials which are opportunistic omnivores, with a diet consisting mainly of birds and carrion. Eymann et al. [162] were not able to observe *N. caninum* antibodies in 142 common brushtail possums (*Trichosurus vulpecula*) from urban Sydney, Australia. The main diet of the common brushtail possums is eucalyptus leaves, but they also eat small mammals. 

### 4.4. *Neospora caninum* in Aquatic (Marine and River) Species

Few epidemiological studies have been performed in either marine or river aquatic mammals (Table 7). 

NAT antibodies were found in seven marine mammal species, namely, walruses (*Odobenus rosmarus*), sea otters (*Enhydra lutris*), harbor seals (*Phoca vitulina*), sea lions (*Zalophus californianus*), ringed seals (*Phoca hispida*), bearded seals (*Erignathus barbatus*), and bottlenose dolphins (*Tursiops truncatus*) in the USA [153]. One killer whale (*Orcinus orca*) showed antibody binding to *N. caninum* antigens by WB, but could not be confirmed by agglutination or PCR [163]. The presence of *N. caninum* antibodies has also been reported in free-ranging seal populations in Hokkaido Island, Japan [154]. Antibodies against NcSAG1t were also detected from Kuril harbor seals (*Phoca vitulina stejnegeri*) and spotted seals (*Phoca largha*) in Japan [154]. 

Recently, antibodies against *N. caninum* have also been reported in sea otters (*Enhydra lutris neresis*) [155], which are a very susceptible species to infection by protozoa such as *T. gondii* or *Sarcocystis neurona*. Titres to *N. caninum* >320 were observed in 4 of 16 sea otters by IFAT [155], and a study detected *N. caninum* DNA in European or Eurasian otters (*Lutra lutra*) (1 of 24, 4.2%) [64]. The positive otter was from a coastal area of Ireland [64]. These authors did not find antibodies in any otters, which may indicate that seroprevalence levels are underestimated for this species. The European otter is threatened across its range and the possible exposure to disease through food and habitat choice warrants more study [64]. In Alaska, antibodies against *N. caninum* were not found in any of 40 river otters (*Lontra canadensis*) [164], and previous studies in European otters were also negative. Five European otters tested for *N. caninum* antibodies by Sobrino et al. [40] and a single otter tested in another study by molecular methods [77] were all negative. In contrast, *T. gondii* infection in European otters in Spain was high, 100% of 6 Eurasian otters [165]. These results could indicate higher water contamination by *T. gondii* oocysts than by *N. caninum* in the areas where the otters were analyzed in Spain [40]. 

Infection of marine species by *N. caninum* indicates that the sea environment has been contaminated with protozoa [154], and these findings suggest that marine mammals might serve as IHs of *N. caninum*. However, more studies are needed to confirm this suggestion and to rule out the possibility of serological cross-reactivity with unidentified organisms. If these marine mammals are indeed confirmed to be IHs of the parasite, then many fundamental questions will arise regarding its transmission through the sea [2]. 

## 5. Control Measures for Neosporosis in Domestic Cycle Taking into Account the Sylvatic Cycle 

At present there is no effective treatment or vaccine for bovine neosporosis. In fact, there is very little information regarding treatments for cattle in field conditions [166] along with neither chemotherapy, or an efficient vaccine for bovine neosporosis [4, 167, 168]. In dogs, although some treatments can be performed to diminish the impact of the infection, they do not eradicate infection and there is no indication that treatment is able to stop oocyst elimination by dogs or wild canids. 

Now that we must consider the participation of wildlife in the life cycle of *N. caninum*, control measures for neosporosis in domestic animals, particularly on cattle farms, where measures have been previously focused on dogs and cattle, could become more complicated [68]. The exact role of birds, rodents, marine mammals, and other wildlife in the life cycle and transmission of *N. caninum* needs to be confirmed and better understood. 

The control measures advised for cattle farms are directed to decrease vertical transmission and to minimize within-herd seroprevalence. Thus, the serostatus of cattle needs to be closely monitored, and to achieve this, discontinued breeding with offspring from seropositive cows and use of beef bull semen in seropositive cows to reduce seroprevalence of infection and abortion in the herds have been recommended [169].

In addition, these measures should be complemented with control of horizontal transmission and in this regard control of wildlife needs to be considered. Although each farm situation is unique, those with intensively farmed cattle should consider the application of strict dog-wild canid management measures in the herds, and where possible, canids, particularly dogs, should not be around farms. Such measures could include erection of canid-proof fences around silage piles, baled hay, and other feedstuffs that are kept outdoors [68]. Dead livestock and the offal from home slaughter domestic animals should be disposed of in a manner that prevents consumption by domestic and wild canids [68]. An interesting consideration is that, in some circumstances, dogs may help to repel wild canids, as has been observed with coyotes [170]. Barling et al. [170] observed decreased odds of seropositivity associated with using a cattle-working dog (most likely due to the deterrence of wild canids) and with using a self-contained cattle feeder [170]. In any event, wild canids and dogs should not have access to abortions, placenta or bovine tissue. Since rodents and birds are also infected, biosecurity control of rodents and wild birds should also be implemented.

In agreement with other authors, we do not recommend a policy of coyote, wolf, or wild canid eradication because of these animals' ability to adapt to changing conditions, to rapidly rebound from reduced population density, and also because of public sentiment [19, 37].

Another measure is to educate hunters not to leave offal from hunted animals in the field. It is quite common that when wildlife is hunted, carcasses are field dressed and the offal is left behind and so is available for scavengers, such as wild canids and feral and domestic dogs in rural areas. 

All these strategies will need to be performed on a long-term basis. 

In summary, although in the last few years much research has been performed on the role of wildlife in *N. caninum* infection, many questions remain unanswered. The demonstration of a *N. caninum* sylvatic cycle does not only help to understand its epidemiology in wildlife but also to clarify the epidemiology of the infection in domestic species and how to improve control measures. The prevalence of infection in wild species in an area could also reflect a high risk of acquiring infection for domestic animals [171]. The research possibilities involving wildlife are numerous, and future studies will hopefully answer many of the current questions on *N. caninum* in wild animals.

## Figures and Tables

**Table 1:** Geographical distribution and species of carnivores in the wild seropositive to *Neospora caninum* antibodies and/or DNA detection.

Species	Seroprevalence studies		DNA detection studies	
	Country	References	Country	References
Canids				
Coyote	USA	[19, 60]		
(*Canis latrans*) confirmed DH [19]	USA-Alaska	[65]		
	Canada	[66]		
Australian dingo (*Canis lupus dingo*) confirmed DH [20]	Australia	[67]		
Gray wolf (*Canis lupus lupus*) confirmed DH [21]	North America			
	USA	[68, 69, 99]		
	USA-Alaska	[65]		
	Europe			
	Spain	[40]		
	Scandinavia	[70]		
	Other areas			
	Israel	[71]		
Golden Jackal (*Canis aureus*)	Israel	[71]		
Aboriginal and feral wild dogs (some hybrid with dingoes)	Australia	[51]		
African wild dogs (*Lycaon pictus*)	Kenya	[82]		
Red fox (*Vulpes vulpes*)	Europe			
	UK	[67, 79, 100]	Spain	[76]
	Ireland	[52, 64, 101]	Czech Republic	[77]
	Belgium	[61]	Belgium	[78]
	Germany (fur farm)	[102]	Ireland	[64]
	Poland	[80]	UK	[54]
	Hungary	[103]		
	Spain	[40, 81]		
	North America			
	Canada	[66]		
	Other areas			
	Israel	[71]		
Culpeo fox (*Dusicyon culpaeus*)	Argentina	[104]		
South American gray fox (*Dusicyon griseus*)	Argentina	[104]		
North American gray fox (*Urocyon cinereoargenteus*)	USA	[62]		
Azara's fox (*Lycalopex gymnocercus*)	Brazil	[105]		
Crab-eating fox (*Cerdocyon thous*)	Brazil	[105]		
Spotted hyena (*Crocuta crocuta*)	Kenya	[32]		
Raccoon dog (*Nyctereute procyonoides*)	Korea	[106]		

Mustelids				
Stone martin (*Martes foina*)	Spain	[40]		
Pine martin (*Martes martes*)	Spain	[40]		
Eurasian badger (*Meles meles*)	Spain	[40]	UK	[54]
Polecat (*Mustella putorius*)	Spain	[40]	UK	[54]
Ferret (*Mustela furo *or* M. putorius furo*)			UK	[54]
American mink (*Neovison vison*)	Ireland	[64]	Ireland	[64]
			UK	[54]

Viverrids				
Common genet (*Genetta genetta*)	Spain	[40]∗		

Herpetids				
Egyptian mongoose (*Herpestes ichneumon*)	Spain	[40]∗		
		[85]		

Felids				
Feral cat (*Felis silvestris catus*)	Spain	[86]		
Eurasian wild cat (*Felis silvestris silvestris*)	Spain	[40]		
Iberian lynx (*Lynx pardinus*)	Spain	[40]		
Cheetah (*Acinonyx jubatus*)	Kenya	[32]		
	Namibia	[87]		
Lion (*Panthera leo*)	South Africa	[87]		
	Kenya	[32]		

Wild carnivore-scavenger birds				
Common raven (*Corvus corax*)	Spain	[93]		
Magpies (*Pica pica*)			Spain	[92]
Common buzzard (*Buteo buteo*)			Spain	[92]

Procyonids				
Raccoon (*Procyon lotor*)	USA	[107]	USA	[108]∗∗

Carnivore marsupials				
Fat-tailed dunnart (*Sminthopsis crassicaudata*)	Australia	[38]	Australia	[38]

∗Positive only by cELISA, not confirmed by NAT and/or IFAT.

∗∗Detected by histologic, immunohistochemical, and molecular methods in the brain of a free-ranging raccoon (*Procyon lotor*) during a canine distemper virus (CDV) outbreak.

**Table 2:** Wild carnivore species positive for *Neospora caninum* antibodies in zoo collections and/or in captivity worldwide.

Species	Positive seroprevalence studies	
	Country	References
Eurasian or European wolf (*Canis lupus lupus*)	Czech Republic	[84]
	Brazil	[95]
Maned wolf (*Chrysocyon brachyurus*)	Brazil	[50, 95, 109]
	Czech Republic	[84]
Chiloe fox (*Pseudalopex fulvipes*)	Chile	[110]
	Czech Republic	[84]
Cheetah (*Acinonyx jubatus*)	Czech Republic	[84]
Jaguarundi (*Puma yagouaroundi* syn. *Herpailurus yagouaroundi*)	Czech Republic	[84]
	Brazil	[95]
Eurasian lynx (*Lynx lynx*)	Czech Republic	[84]
Indian lion (*Panthera leo goojratensis*)	Czech Republic	[84]
Fisher (*Martes pennanti*)	Czech Republic	[84]
Fennec (*Vulpes zerda*)	Czech Republic	[84]
Ocelot (*Leopardus pardalis*)	Brazil	[95]
Little spotted cat (*Leopardus tigrinus*)	Brazil	[95]
Jaguar (*Panthera onca*)	Brazil	[95]
Puma (*Puma concolor*)	Brazil	[95]
Tiger (*Panthera tigris*)	Brazil	[95]
Pampas cat (*Oncifelis colocolo*)	Brazil	[95]
Caracal (*Caracal caracal*)	Brazil	[95]
Serval (*Letailurus serval*)	Brazil	[95]
Lion (*Panthera leo*)	Senegal	[111]
	Brazil	[95]
Fishing cat (*Prionailurus viverrinus*)	Brazil	[95]
Bush dog (*Speothos venaticus*)	Brazil	[94, 95]
Crab-eating fox (*Cerdocyon thous*)	Brazil	[95]
Hoary fox (*Pseudalopex vetulus*)	Brazil	[95]
Blue foxes (*Alopex lagopus*)	China (farm-bred)	[96]∗

∗Histopathological and immunohistochemical examinations.

**Table 3:** Geographical distribution and species of herbivores in the wild seropositive to *Neospora caninum*, DNA detection, and/or isolation of *N. caninum. *

	Positive seroprevalence		Positive DNA detection		Positive isolation	
Species of wild ruminants	studies		studies		studies	
	Country	References	Country	References	Country	References
North America						
White-tailed deer (*Odocoileus virginianus*)	USA	[13, 58, 68, 113, 114]			USA	[36]
	Mexico	[112]				
	Canada	[115]				
Bison (*Bison bison*)	USA-Alaska	[69]				
Musk ox (*Ovibos moschatus*)	USA-Alaska	[69]				
Moose (*Alces alces*)	USA	[69]				
	USA-Alaska	[65]				
	Canada	[115]				
Caribou (*Rangifer tarandus*)	USA	[69]				
	USA-Alaska	[65]				
	Canada	[115]				
Elk (*Cervus canadensis*)	Canada	[115]				
						
Black-tailed deer (*Odocoileus hemionus columbianus*)	USA	[116]	USA	[57]∗∗		
Mule deer (*Odocoileus hemionus hemionus*)	USA	[116]				
Pampas deer (*Ozotoceros bezoarticus*)	Brazil	[117]				

Europe						
Red deer (*Cervus elaphus*)	Italy	[59, 118]				
	Spain	[48]				
	Poland	[119]				
	Greece	[120]				
Chamois (*Rupicapra rupicapra*)	Italy	[59, 118, 121]				
	Italy	[118, 121]	Belgium	[78]		
Roe deer (*Capreolus capreolus*)	Spain	[48, 122]				
	Czech Republic	[123]^a^				
	Belgium	[78]				
	Sweden	[124]				
						
Alpine ibex (*Capra ibex*)	Italy	[118]∗				
						
Spanish ibex (*Capra pyrenaica hispanica*)	Spain	[125]				
European mouflon (*Ovis musimon*)	Czech Republic	[123]^a^				
Fallow deer (*Dama dama*)	Czech Republic Poland	[123]^a^ [126]				
Barbary sheep (*Ammotragus lervia*)	Spain	[48]				
European bison (*Bison bonasus bonasus *L.)	Poland	[127, 128]			Poland	[127]
Moose (*Alces alces*)	Sweden	[124]^b^				

Asia						
Tarim red deer (*Cervus elaphus yarkandensis*)	China	[129]				
Africa						
Zebra (*Equus burchelli*)	Kenya	[32]				
Eland (*Taurotragus oryx*)	Kenya	[32]				
African buffalo (*Syncerus caffer*)	Kenya	[32]				
Thomson's gazelle (*Eudorcas thomsonii*)	Kenya	[32]				
Impala (*Aepyceros melampus*)	Kenya	[32]				

∗Cited by [118].

^a^Included some animals from hunting farms.

^b^Positive results by iscom ELISA, negative by WB.

∗∗Systemic neosporosis.

**Table 4:** Wild herbivore species positive for *N. caninum* antibodies or DNA detection in zoo collections and/or in captivity worldwide.

Species	Positive seroprevalence studies	
	Country	References
Ruminants		
Eld's deer (*Cervus eldi siamensis*)	France	[56]∗
Antelope (*Tragelaphus imberbis*)	Germany	[98]∗
Blackbuck (*Antilope cervicapra*)	Czech Republic	[84]
Lechwe (*Kobus leche*)	Czech Republic	[84]
African buffalo (*Syncerus caffer caffer*)	Czech Republic	[84]
Eland (*Taurotragus oryx*)	Czech Republic	[84]
European bison (*Bison bonasus*)	Czech Republic	[84]
Sitatunga (*Tragelaphus spekei gratus*)	Czech Republic	[84]
Pere David's deer (*Elaphurus davidianus*)	Czech Republic	[84]
Thorold's deer (*Cervus albirostris*)	Czech Republic	[84]
Eastern elk (*Cervus elaphus canadensis*)	Czech Republic	[84]
Vietnam sika deer (*Cervus Nippon pseudaxis*)	Czech Republic	[84, 123]
Brocket deer (*Mazama spp.*)	Brazil	[130]
Musteloidea		
Red pandas (*Ailurus fulgens*)	China	[131]
Proboscidea		
Elephants *(Elephaus maximus indicus) *	Thailand	[132]
Perissodactyla		
	South Africa	[133]∗
White rhinoceros (*Ceratotherium simum*)	Thailand	[134]∗
	Australia	[135]∗
Herbivore marsupials		
Parma wallaby (*Macropus parma*)	Austria	[136]∗

∗Clinical cases.

**Table 5:** Geographical distribution and species of wild lagomorphs, rodents, and insectivorous species seropositive to *Neospora caninum* antibodies and/or DNA detection*. *

Species	Positive seroprevalence studies		Positive DNA detection studies	
	Country	References	Country	References
Lagomorphs				
Wild rabbit (*Oryctolagus cuniculus*)	Egypt	[137]	UK	[138]
Iberian Hare (*Lepus granatensis*)	Spain	[48]^+^		
Brown hare (*Lepus europaeus*)	Hungary-Slovakia	[139]		
	Austria-Czech Republic	[140]		

Rodents				
Rat (*Rattus norvegicus*)	Grenada-West Indies Taiwan	[141] [142]	Italy	[55]
House mouse (*Mus musculus*)	USA UK	[141] [143]	Italy Australia	[55] [144]
Field mouse (*Apodemus sylvaticus*)	UK	[143]	Italy	[55]
Capybara (*Hydrochaeris hydrochaeris*)	Brazil	[145, 146]	Brazil	[147]
Vole (*Microtus arvalis*)			Austria	[148]
Water vole (*Arvicola terrestris*)			Austria	[148]
Rock squirrel (*Spermophilus variegatus) *			Mexico	[149]
Harvest mouse (*Micromys minitus*)			The Netherlands	[150]

Insectivorous				
Common shrew (*Sorex araneus*)			The Netherlands	[150]
White-toothed shrews (*Crocidura russula*)			The Netherlands	[150]

^+^cELISA positive results not confirmed by IFAT due to lack of sample.

**Table 6:** Geographical distribution and species of wildlife omnivorous seropositive to *Neospora caninum. *

Species	Positive seroprevalence studies	
	Country	References
Omnivorous-suidae		
Wild boar (*Sus scrofa*)	Czech Republic	[151]
	Spain	[48]
Warthog (*Phacochoerus aethiopicus*)	Kenya	[33]
Omnivorous-marsupials		
South American opossum (*Didelphis marsupialis*)	Brazil	[152]

**Table 7:** Geographical distribution and species of aquatic (marine and river species) in the wild seropositive to *Neospora caninum* antibodies.

Species marine and river mammals	Seroprevalence studies	
	Country	References
Walrus (*Odobenus rosmarus*)	USA	[153]
Sea lion (*Zalophus californianus*)	USA	[153]
Ringed seal (*Phoca hispida*)	USA	[153]
Bearded seal (*Erignathus barbatus*)	USA	[153]
Harbor seal (*Phoca vitulina*)	USA	[153]
Ribbon seal (*Phoca fasciata*)	USA	[153]
Spotted seal (*Phoca largha*)	USA	[153]
	Japan	[154]
Kuril harbor seal (*Phoca vitulina stejnegeri*)	Japan	[154]
Bottlenose dolphin (*Tursiops truncatus*)	USA	[153]
Sea otter (*Enhydra lutris neresis*)	USA	[153, 155]
European otter (*Lutra lutra*)	Ireland	[64]∗

∗DNA detection.

## Abbreviations

95% CI: 95% confidence interval
DAT: Direct agglutination test
DH: Definitive host
DNA: Deoxyribonucleic acid
ELISA: Enzyme linked immunosorbent assay indirect
cELISA: Competitive ELISA
iscom ELISA: ELISA in which extracted tachyzoite proteins incorporated into immunostimulating complexes (iscoms) which are used as coating antigen
NcSAG1t ELISA: ELISA in which recombinant surface antigen 1 of *N. caninum* (NcSAG1) is used as antigen
IH: Intermediate host
IFAT: Indirect immunofluorescent antibody test
IUCN list: International Union for the Conservation of Nature and Natural Resources red list of threatened species
KO mice: Gamma interferon gene knockout mice
NAT: *Neospora* agglutination test
PAS stain: Periodic acid-Schiff stain
PCR: Polymerase chain reaction
p.i.: Postinfection
TG-ROC: Two-graph receiver-operating characteristic
WB: Immunoblotting or Western blot
WTD: White-tailed deer.
